# Preliminary Findings that a Targeted Intervention Leads to Altered Brain Function in Children with Fetal Alcohol Spectrum Disorder

**DOI:** 10.3390/brainsci8010007

**Published:** 2017-12-28

**Authors:** Kelly Nash, Sara Stevens, Hayyah Clairman, Joanne Rovet

**Affiliations:** 1 Psychiatry Department, The Hospital for Sick Children, Toronto, ON M5G1X8, Canada; 2kellynash@gmail.com; 2 Holland Bloorview Kids Rehabilitation Hospital, Toronto, ON M4G1R8, Canada; sstevens@hollandbloorview.ca; 3 Bloorview Research Institute, Holland Bloorview Kids Rehabilitation Hospital, Toronto, ON M4G1R8, Canada; 4 Neurosciences and Mental Health Program, The Hospital for Sick Children, Toronto, ON M5G1A0, Canada; hayyah.clairman@sickkids.ca; 5 Department of Pediatrics, University of Toronto, Toronto, ON M5G1X8, Canada; 6 Psychology Department, University of Toronto, Toronto, ON M5S3G3, Canada

**Keywords:** fetal alcohol spectrum disorder, self-regulation training, executive functioning, inhibitory control, fMRI, Alert^®^ Program for Self-Regulation, neural correlates

## Abstract

Children with fetal alcohol spectrum disorder (FASD) exhibit behavioral dysregulation, executive dysfunction, and atypical function in associated brain regions. Previous research shows early intervention mitigates these outcomes but corresponding brain changes were not studied. Given the Alert^®^ Program for Self-Regulation improves behavioral regulation and executive function in children with FASD, we asked if this therapy also improves their neural functioning in associated regions. Twenty-one children with FASD aged 8–12 years were randomized to the Alert^®^-treatment (TXT; *n* = 10) or waitlist-control (WL; *n* = 11) conditions. They were assessed with a Go-NoGo functional magnetic resonance imaging (fMRI) paradigm before and after training or the wait-out period. Groups initially performed equivalently and showed no fMRI differences. At post-test, TXT outperformed WL on NoGo trials while fMRI in uncorrected results with a small-volume correction showed less activation in prefrontal, temporal, and cingulate regions. Groups also demonstrated different patterns of change over time reflecting reduced signal at post-test in selective prefrontal and parietal regions in TXT and increased in WL. In light of previous evidence indicating TXT at post-test perform similar to non-exposed children on the Go-NoGo fMRI paradigm, our findings suggest Alert^®^ does improve functional integrity in the neural circuitry for behavioral regulation in children with FASD.

## 1. Introduction

Fetal Alcohol Spectrum Disorder (FASD) is the most preventable cause of intellectual disability worldwide [1] affecting as many as 4% of newborns in North America [2] with much higher prevalence in countries such as Italy, South Africa, and Ireland [3,4,5], as well as among aboriginal youth in Australia, U.S., and Canada [6,7,8]. FASD is the umbrella term used to denote the conditions arising from prenatal alcohol exposure (PAE), primarily Fetal Alcohol Syndrome (FAS), partial FAS (pFAS), and Alcohol Related Neurodevelopmental Disorder (ARND). Of these, FAS is characterized by a triad of features that includes a dysmorphic face, abnormal growth, and neurobehavioral impairments [9]; pFAS is similar but with fewer or less severe physical features [10]. In contrast, ARND accounting for most FASD cases has only neurobehavioral manifestations and no discernible physical characteristics [11]. Across subtypes, children with FASD display reduced IQ [12,13,14], poor academic achievement [15,16,17] especially in math [18], and significant disabilities in language, memory, visuospatial, attention, and executive functioning areas [19,20,21,22,23,24,25,26,27]. Within the executive function domain, their impairments reflect poor planning and decision-making and difficulties in working memory and inhibitory control [27,28,29,30,31,32,33]. Research with various magnetic resonance imaging (MRI) techniques has also shown they exhibit atypical brain development [34], as evidenced by their smaller than normal global and regional brain volumes [35,36]; reduced size of caudate, hippocampus, and corpus callosum [21,37,38]; and abnormalities in cortical thickness and surface area [39,40,41,42], gyrification [43,44] and white matter (WM) tract formation [45,46,47]. Furthermore, studies using functional magnetic resonance imaging (fMRI) describe abnormal neural activation patterns on paradigms assessing working memory, visual search, number processing, and attention skills [48,49,50,51,52,53], as well as inhibitory control [54,55,56,57].

FASD is also associated with a broad array of adaptive difficulties [58,59], particularly poor self-regulation [60]. Most infants are severely dysregulated [61,62,63,64], a trait that continues into adulthood [65] often leading to trouble with the law [66]. Among children with FASD, social and emotional problems are endemic with many receiving psychiatric diagnoses [67,68,69,70,71], typically attention deficit hyperactivity disorder and conduct disorder [72,73]. In addition, most adults born to women with heavy alcohol consumption during pregnancy experience a major mental health problem [74] such as depression and substance abuse [75] and have a high suicide risk [76]. Not surprisingly, FASD poses an immense burden to the individuals and their families [77,78], as well as a high cost to society [79,80,81].

Despite consensus that children with FASD need early intervention to prevent the later adverse consequences [66], only a handful of empirically validated therapies surprisingly exist for this population [60,82,83,84], possibly because most treatments lack the necessary specificity [85]. According to Kodituwakku [60], for a therapy to be effective, it must target a core deficit of FASD and because behavioral regulation difficulties, including poor impulse control are so pervasive in this population, a reasonable approach would be to focus on these impairments. One promising therapy is the Alert^®^ Program for Self-Regulation [84,86], which trains children to recognize if and when their arousal levels are abnormal and supplies them ways for modifying these. Research on Alert^®^ has shown benefits for behavioral regulation in children with emotional problems [87] and, more importantly, improved inhibitory control, emotion regulation, and organizational skills in children with FASD [88,89,90]. Currently, we asked if Alert^®^ also leads to altered brain functioning in children with FASD and evaluated this via an inhibitory-control fMRI paradigm.

In the present paper, we provide preliminary evidence on a sample of FASD children who either underwent the Alert^®^ program and were tested before and after it or were assigned to a waitlist-control condition and were tested twice over a comparable period of time. A Go-NoGo inhibitory-control paradigm based on the Whack-a-Mole game was used. Previous fMRI studies with this paradigm found children with FASD (also children with heavy PAE) showed abnormal neural activation patterns, which included (a) greater activation of prefrontal (viz., left medial and right middle frontal gyri), right parietal (viz., inferior parietal lobule, supramarginal gyrus), left temporal (middle temporal gyrus), and left anterior cingulate and (b) less activation of the right caudate and right occipital lobe (middle occipital gyrus) [54,55] than in unexposed typically developing controls. On this basis, we hypothesized that children with FASD treated with Alert^®^ would show changes in these regions in the direction of non-exposed controls while brain function would remain unchanged in untreated FASD children.

## 2. Materials and Methods

### 2.1. Participants

The initial sample included 25 8- to 12-year olds who received a positive diagnosis at either the FASD Follow-up Clinic at The Hospital for Sick Children (SickKids; *n* = 16) or another FASD diagnostic facility in southern Ontario (*n* = 9). At SickKids, the diagnostic process involved an extensive assessment by a team consisting of a pediatrician, psychologist, psychometrist, and speech language pathologist. At this site, the Canadian Diagnostic Guidelines system [11] was used to formulate the diagnosis and only FAS and ARND classifications were considered. The other cases were seen either by single practitioners or in accredited FASD clinics, where the Diagnostic Guide for Fetal Alcohol Syndrome and Related Conditions: The 4-Digit Diagnostic Code (2nd ed.) from the University of Washington [91] was used. At these facilities, children could receive the pFAS designation, as well as FAS and ARND. The current sample included 21 children with ARND (16 from SickKids, 5 elsewhere), 4 with pFAS (all elsewhere), and none with FAS.

Recruitment of SickKids’ cases involved selecting children from clinic files and sending their parents/caregivers a letter describing the project and seeking participation in it. The other cases were recruited via advertising at local FASD support groups or web postings (e.g., FASWorld). All interested parents/caregivers were asked to contact our study coordinator for a brief prescreening telephone interview in order to establish eligibility and arrange scheduling. Only included were children with heavy PAE exposure, based on testaments of close relatives caring for them or Children’s Aid Society records documenting mothers’ alcohol abuse during pregnancy and if alcohol was the primary prenatal exposure. Children with significant head injuries requiring hospitalization or wearing braces (a contraindication to MRI testing) were excluded.

### 2.2. Design and Procedures

The study involved a randomized treatment/control design with pre-test and post-test measurements using fMRI. All children except four were randomly assigned either to an immediate-treatment (TXT) or to waitlist-control (WL) group. Exceptions were two pairs of siblings whose parents explicitly requested both children treated concurrently; one set of siblings was randomized to the TXT group and the other to the WL group.

Pre-testing involved both clinical testing and MRI scanning over the course of two days approximately one week apart (range = 2 to 30 days) with scanning on day two. Also administered at pretest only was the Wechsler Abbreviated Scale of Intelligence (WASI) with IQ based on the two-subtest version [92]. Post-testing took place on a single day approximately 18 weeks after pre-testing (mean ± SD = 17.9 ± 1.7 weeks) and involved a selection of the clinical tests at pre-test but the exact same scanning procedures. Alert^®^ was provided to the TXT group between pre-test and post-test sessions and to the WL group on study completion. Although every effort was made to keep relevant personnel blinded to group status, this may have been realized for some children given their different frequencies of coming to the lab.

At the end of the study, families were compensated for their travel expenses; children were given a CD containing images of their own brain scan and several movie passes. Within two months of post-testing, parents/caregivers were mailed a report describing their child’s test results and invited back for a 1-h feedback session to discuss this. Each child’s physician was also sent a copy of the neuroradiological report.

All procedures were approved by the Research Ethics Board at SickKids (file # 100014076, approved on October 2008). The study was also registered with ClinicalTrials.gov Protocol Registration and Results System (Registration #NCT02456767).

### 2.3. The Intervention

Two advanced-level graduate students (KN, SS) administered the Alert^®^ Program for Self-Regulation [84,86]. Both received formal training at an official Alert^®^ site and ongoing supervision from a clinical psychologist. The therapy was provided in the SickKids Psychology Department in a dedicated room equipped with Alert^®^ program-specified materials (e.g., floor mats, therapy balls, inner tubes, pillows, tent, caterpillar tunnel, and manipulanda) and devoid of unnecessary visual and auditory distractors.

Alert^®^ is based on an analogy of car engine running at different speeds with children being taught to recognize when their engines run too quickly or too slowly and how to adjust them to run “just right”. The program involves 12 individualized 1.5-h weekly sessions that provide program-specified activities focusing on emotion sensitization and recognition, behavioral regulation, and social problem solving. Sessions are divided into three stages. In Stage 1, children are familiarized with and learn to label their engine speeds; in Stage 2, they are given strategies for changing their engine speeds; and in Stage 3, they are given training in how and when to use the different strategies outside the therapy venue. The program spanned on average ~14 weeks to allow for illness and vacations.

### 2.4. Neuroimaging Task, Procedures, and Image Analysis

**Figure 1:**
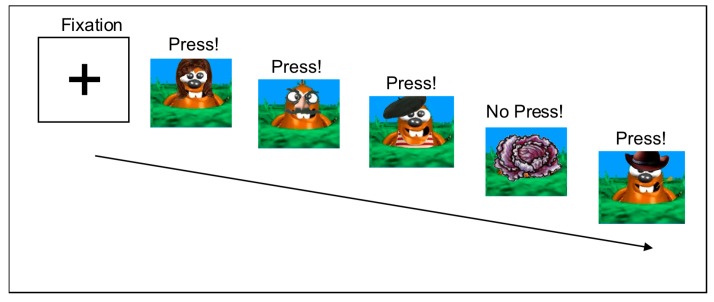
Go-NoGo Task Stimuli. Instructions were to press for all moles and not press for vegetables. Stimuli were presented for 250 ms with a jittered interstimulus index ISI averaging 1250 ms (range = 970–1530 ms).

The fMRI paradigm was based on the “Whack-a-Mole” game [93,94]. Stimuli consisted of 12 distinct cartoon moles or a colorful garden vegetable (see Figure 1) derived from the Sackler Institute website [95] and shown on a black background. A laptop computer programmed with Presentation^®^ software version 14.1 (www.neurolabs.com) served to present the task. Children using an MRI-compatible button box pressed a response key with their dominant hand whenever a mole appeared and refrained from responding for a garden vegetable. Accuracy and reaction time (RT) data were recorded on-line.

Initially, all participants received familiarization with the Whack-a-Mole game outside the scanner, at which time instructions on participating in the scanner using pictures and imitations of scanner sounds were also given. Within the scanner, testing was provided via a laptop computer connected to Resonance Technology Magnetic Resonance (MR)-compatible goggles. An event-related design was used to present the task that consisted of 6 runs of 60 trials (45 Go trials (moles) and 15 NoGo trials (vegetables)). Presentation time was 250 ms and the interstimulus interval was jittered between 970 and 1530 ms (average = 1250 ms), for a total run time of ~90 s. Total task duration was 9 min.

Imaging was conducted on a 1.5 Tesla GE Medical Systems Signa Excite magnet, the only one at SickKids available for research purposes when our study began. An 8-channel head coil was used. Anatomical images were acquired via a T1-weighted inversion recovery prepared fast spoiled gradient echo image (Repetition Time (TR )= 10.37 ms; Echo Time (TE )= 4.26 ms; inversion time = 400 ms; flip angle = 20°; field of view (FOV) = 24 cm; slice thickness = 1.5 mm reconstructed at 1.0 mm^3^). Functional data were obtained using echo-planar imaging (TR = 2 s, 25 slices, FOV = 240 mm, 64 × 64 matrix, resulting in a voxel size of 3.75 × 3.75 × 5 mm^3^) and acquired axially with an interleaved design. Each stimulus event was modeled in Statistical Parametric Mapping version 5 (SPM5) using a canonical hemodynamic response function beginning at the onset of each trial.

Preprocessing involved slice-time correction, realignment, and screening for excessive motion. For each run, movement exceeding ±2 mm and/or ±2 degrees rotation from baseline was noted and all runs with large motion spikes were removed. Analyses were based only on data from runs free of excessive motion. Further preprocessing involved (i) co-registering to the participant’s T1-weighted structural image; (ii) normalizing data to the Montreal Neurological Institute (MNI) template; (iii) resampling at a 4 × 4 × 4 mm^3^ voxel size; and (iv) smoothing using a Gaussian kernel of 8 mm full-width half maximum. Only correct response trials were analyzed and runs with accuracy below 20% were discarded. The image analyst was blinded to group status.

Two series of analyses were performed: a whole-brain and a region-of-interest (ROI) analysis examining blood oxygen-level dependent (BOLD) activity in the prefrontal cortex or caudate, as per [54]. The prefrontal cortex was defined manually to include all tissue anterior to the genu of the corpus callosum [54] and the caudate was defined using the caudate mask in MarsBaR (MARSeille Boîte À Région d’Intérêt) Automatic Anatomical Labelling (AAL) ROI package in SPM5 [96].

### 2.5. Statistical Approach and Analyses

Demographic data were analyzed via t-tests and chi-square (*χ*^2^) using the Statistical Package for the Social Sciences (SPSS) software version 22 (IBM, Armonk, NY, USA). For performance data (accuracy, RT), results were analyzed in SPSS version 24 (IBM, Armonk, NY, USA) via repeated measures Group × Time (pre-test/post-test) × Condition (Go-NoGo) Analyses of Variance (ANOVA) as well as simple ANOVAs or Analyses of Covariance (ANCOVA) to decipher significant effects. The *p-*value was set at 0.05 using a one-tailed test given our stated hypotheses.

The imaging data were analyzed in SPM5 with the NoGo minus Go difference as the contrast-of-interest. First, each subject’s pre-test and post-test contrast data were modeled using a fixed-effects method. Next, groups were compared at pre-test and post-test using independent samples *t-*tests, with age and overall accuracy as covariates of no interest. Group differences in change over time were analyzed using a flexible (repeated measures) factorial model. For all significant interactions, percent signal-change analyses were conducted by drawing a 6 mm radius sphere at the interaction’s point of peak activation and measuring the signal within the sphere. Groups were compared for signal-change differences using repeated-measures ANOVAs in SPSS version 23 (IBM, Armonk, NY, USA).

For whole-brain and prefrontal-ROI data, the extent was set at 10 contiguous voxels. Given the caudate’s smaller size, an extent of only 5 contiguous voxels was allowed, consistent with [54] where the extent was set at 4 voxels. For neuroimaging analyses, results were thresholded at *p* < 0.05 level using the false discovery rate (FDR). Because of the preliminary nature of this study, results were also explored using a small-volume correction (SVC) at the peak value, with significance set at *p* < 0.01.

## 3. Results

### 3.1. Demographic Data

Table 1 presents the demographic data for the 25 FASD participants initially randomized to the two conditions. TXT and WL differed only on secondary drug exposure rates (*χ^2^* (1) = 3.8, *p* < 0.05), which was higher in the TXT group. As also shown in Table 1, there were no group differences in age, sex, socioeconomic status (SES), attention-deficit hyperactivity disorder (ADHD) or other psychiatric diagnoses, stimulant medications, or IQ. Groups also did not differ in elapsed time between sessions (TXT = 125.8 ± 17.8 days; WL = 124.9 ± 7.0 days). Because most biological mothers were not available to affirm secondary-exposure rate reports, we lacked confidence in using this as a covariate.

### 3.2. Behavioral Results

Four children were eliminated from the neuroimaging analyses: two were from the TXT group who refused to enter the scanner due to anxiety, one was from WL group who received a dental implant between sessions, and another was from WL group who had an excessive motion artifact. Thus, the final sample consisted of 10 TXT (5 males, 1 left handed) and 11 WL (6 males, 2 left handed), who did not differ in demographics from the four cases eliminated.

**Figure 2:**
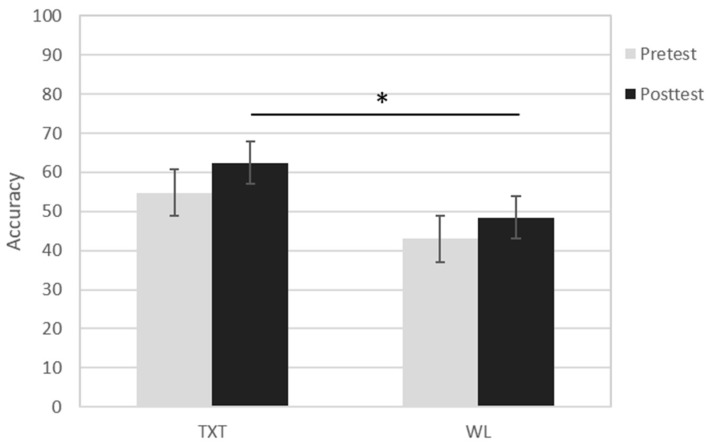
Mean NoGo accuracy at pre-test and post-test in treatment (TXT) and Waitlist (WL) groups. * signifies *p* < 0.05.

Table 2 presents the Go-NoGo performance data during scanning. Accuracy was generally higher on Go (87.6%; pre-test range = 53 to 99%; post-test range = 57–100%) than NoGo trials (42%; pre-test range = 20 to 100%; post-test range = 27 to 86%). A mixed model Group X Time X Condition ANOVA showed a significant main effect of Trial Type (F(1,20) = 109.5, *p* < 0.001, *η*^2^ = 0.85), due to both groups’ better performance on Go than NoGo trials. Also observed was a significant Group × Trial Type interaction (F(1,20) = 3.80, *p* < 0.066, *η^2^* = 0.60), which reflected the better performance of TXT than WL on NoGo trials (F(1,20) = 6.45, *p* < 0.05, *η*^2^ = 0.25), especially at post-test (F(20) = 3.23, *p* < 0.05) (see Figure 2). To assess whether the post-test difference on NoGo trials reflected the initially better performance of the TXT group, we also reanalyzed the post-test data using pre-test accuracy as a covariate. Results showed the post-test difference remained (F(1,19) = 3.94, *p* < 0.05), signifying a likely impact of the intervention. There were no significant effects for reaction time.

### 3.3. fMRI Results

Number of runs excluded for motion was similar in TXT and WL groups (4.1% and 6.1% respectively). However, TXT had more runs excluded at pre-test, whereas WL had more at post-test (pre-test: TXT = 8.3%, WL = 1.5%; post-test: TXT = 0%, WL = 10.6%). Results revealed no significant effects in either whole-brain or region-of interest analyses with the FDR correction applied. Nevertheless, because of this study’s preliminary nature, uncorrected results using the small-volume correction in SPM5 were also examined.

#### 3.3.1. Whole Brain Results

**Figure 3:**
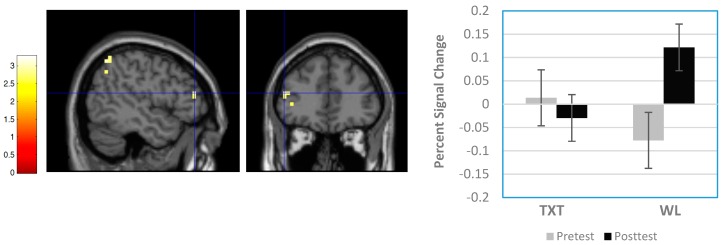
Sample Group × Time interaction result from whole-brain analysis. Panel on left shows sagittal and coronal views of significant cluster in left middle frontal gyrus (Brodmann Area (BA) 46; Montreal Neurological Institute (MNI) coordinates = −48 40 16). Panel on right shows percent signal change differences between groups. Note signal decreased between pre-test and post-test in TXT and increased in WL.

**Figure 4:**
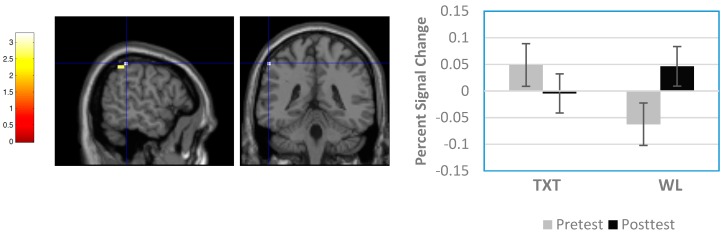
Sample Group × Time interaction result from whole-brain analysis. Panel on left shows sagittal and coronal views of significant cluster in left inferior parietal lobule (Brodmann Area (BA) 40; Montreal Neurological Institute (MNI) coordinates = −56 −40 52). Panel on right shows percent signal change differences between groups. Note signal decreased between pre-test and post-test in TXT and increased in WL.

Six regions showed significant clusters (uncorrected results) differentiating TXT and WL groups. All occurred at post-test; there were no pre-test differences. As shown in Table 3, clusters reflecting significant differences were found in the right medial frontal gyrus (Brodmann Area 8 (BA8)), left frontal subgyral region (white matter), right cingulate gyrus (BA32), left cingulate gyrus (BA24), right putamen, and right superior temporal gyrus (BA22). In each case, activation was greater in WL than TXT. Also observed were three regions involving significant Group × Time interactions: a 26-voxel cluster in the right superior frontal gyrus (BA6), a 14-voxel cluster in the left middle frontal gyrus (BA46), and a 17-voxel cluster in the left inferior parietal lobule (BA40). For these three regions, analysis of percent signal change data indicated significant interactions for all three locations (F(1,19) = 8.29, *p* = 0.010, *η*^2^ = 0.304; F(1,19) = 6.137, *p* = 0.023, *η*^2^ = 0.244; F(1,19) = 5.109, *p* = 0.036, *η*^2^ = 0.210 respectively). Figure 3 and Figure 4, which provide spatial maps and percent signal-change results for the interactions at BA46 and BA40, indicate that activation declined between sessions for TXT and increased for WL. Similar effects were observed for the interaction at BA6.

#### 3.3.2. Region-of-Interest Results

For the frontal ROI, exploratory analyses with the SVC applied identified only one significant effect, a Group × Time interaction reflecting a 14-voxel cluster in the left middle frontal gyrus (BA46; MNI coordinates: −48 40 16; SVC *p*-value = 0.005). As with the whole-brain findings (see Figure 3), this effect reflected reduced activation between sessions for TXT and increased for WL. There were no between-group differences at either session.

There were no effects for the caudate ROI.

## 4. Discussion

The main goal of this study was to ascertain whether children with FASD would show altered brain functioning after an intervention targeting their core deficit of behavioral dysregulation, which includes poor impulse control. We currently contrasted children who received the Alert^®^ Program of Self-Regulation versus those in the waitlist-control group, who received Alert^®^ after study completion. All children were evaluated with an fMRI inhibitory-control paradigm before and after the therapy (TXT group) or over a comparable period of time (WL group). In previous research giving variants of this paradigm to children with FASD (or heavy PAE), results indicated they had atypical fronto-striatal and fronto-parietal engagement relative to unexposed typically developing youth. In particular, the exposed group showed increased activation within prefrontal, cingulate, and parietal regions and decreased within caudate. On this basis, we hypothesized that children with FASD after Alert^®^ would show improved response inhibition as well as functional changes in the same neural regions. Moreover, the observed changes would be in the direction of the non-exposed youth of former studies. However, when we used a stringent criterion for determining significance (viz., the FDR correction), we failed to find any differences between groups and no differential change regions showing over time within regions. Therefore, given the exploratory and unique nature of this study in evaluating brain changes arising from a therapy aimed at a core FASD deficit, we further examined uncorrected data but applied a small-volume correction procedure.

Accordingly, we found a number of significant effects, most as predicted. Specifically, children with FASD after Alert^®^ were better able to withhold a prepotent response than their waitlisted counterparts. Furthermore, neuroimaging results revealed six regions that differentiated the groups at post-test. These were located in the right medial frontal gyrus (BA8), left frontal subgyral region, the right and left cingulate gyri (BA32 and BA24 respectively), right putamen, and right superior temporal gyrus (BA22). In all regions, TXT showed less neural engagement than WL. In addition, we also observed three regions with significant (uncorrected data) interaction effects signifying different patterns of change for the two groups. The three regions were located in the right superior frontal gyrus (BA6), left middle frontal gyrus (BA46, whole-brain and ROI analyses), and left inferior parietal lobule (BA40). In each case, interactions reflected a decline in activation for TXT and an increase for WL at post-test. Given previous studies comparing children with PAE and typically-developing controls on Go-NoGo fMRI paradigms, current findings suggest the treated FASD groups is starting to resemble the children never exposed prenatally to alcohol in those studies [54]. In other words, our findings suggest improved brain functioning, possibly implying more mature neural integrity [97]. Overall, these findings provide initial support for our hypothesis that a therapy targeting a core deficit of FASD will lead to improved brain functioning in these children. However, contrary to expectation, we did not find the WL group remained static but rather showed an increase in activation within the same brain regions at post-test, perhaps reflecting more effort on task repetition in their suboptimally functioning brain regions.

A recent review of the neural cohorts engaged during a response inhibition paradigm such as the Go-NoGo task has suggested a series of distinct brain networks are successively engaged to carry out various requirements of this task [98]. The first is a fronto-striatal-thalamic circuit that primes the motor system to respond. Next is a parietal-premotor circuit that engages inferotemporal and inferior frontal cortices to transform incoming sensory information into action. Finally, the inhibitory control network that activates frontal and parietal regions and deactivates the caudate, cingulate, and premotor cortices is called upon to suppress the tendency to respond. Current findings showing effects in structures belonging to these circuits suggests Alert^®^ does lead to improved integrity within brain regions serving to interpret incoming sensory information and prepare children to execute the proper responses. Furthermore, our finding of effects in the left middle frontal gyrus (BA46), which is known to be important for self-regulation particularly inhibition [99], suggest Alert^®^ has contributed to functional changes in a critical region for this targeted ability.

Our observation that TXT showed less bilateral anterior cingulate activation than WL at post-test is contrary to expectation, particularly in light of recent structural imaging findings showing adolescents with heavy PAE have reduced anterior cingulate cortex surface area and this is correlated with their poorer cognitive control [100]. Since the right anterior cingulate is known to modulate reward value and error detection, it is possible that current findings signify children treated with Alert^®^ were receiving less pleasure from whacking a mole than their untreated counterparts and, instead, were now more focused on whether or not to respond [101]. Alternatively, our findings in right and left anterior cingulate cortices may reflect improved sustained attention [102] and verbal working memory capacity for carrying out the task [101].

We also observed the groups at post-test had different activation of the right superior temporal gyrus (STG), a region that is critical for emotion perception and social cognition [103,104]. Our findings showing less activation in treated FASD children may suggest they switched from focusing on the moles’ facial expressions to the task at hand due to the therapy. It is interesting to note that findings from a recent fMRI study comparing STG functions of typically developing adults versus children indicated the adults engaged the STG more anteriorly than did the children [105]. Since we observed less anterior STG activation in our treated sample following Alert^®^ than Fryer’s PAE group [68], albeit the opposite hemisphere, the current anterior shift may represent a tendency towards more normal or mature neural responding after the training.

We did not observe any group differences or different patterns of change in the caudate, which was previously observed to be less activated in children with PAE than non-exposed controls, and was thought to reflect appropriation of fewer cognitive resources (i.e., less attention) to the task they were given [54]. While current findings suggest no effect of Alert^®^ in this region, and hence no changes in attention, we cannot confirm this until a similar study with typically developing youth is conducted.

Researchers of children with heavy PAE have also found they exhibit greater engagement of the right inferior parietal lobule and right supramarginal gyrus than their unexposed controls on similar paradigms [54,57]. In the current study, we observed that children with FASD after treatment responded similar to the unexposed controls of the other studies but within the left, not the right, parietal lobule (see interaction analysis results). This shift in laterality may reflect the different stimuli and task demands of our study, which used lively highly discriminable images for Go versus NoGo decisions, versus the other studies, which were more perceptually demanding and required differentiating among stimuli on the basis of their shape and size. Alternatively, our findings may suggest a compensatory response with neural reorganization to the opposite hemisphere.

This investigation is to our knowledge the first of its kind to use fMRI for evaluating the benefits at a neural level of a targeted intervention for children with FASD. Nevertheless, our paper does have a number of limitations. First and foremost, our small sample size would have led to low power, thus reducing our ability to detect true effects. Consequently, we obtained no significant effects with the FDR correction and had to resort to examining uncorrected results, which in turn may have increased the reporting of false-positive results. However, we attempted to minimize this using a small-volume-correction procedure and a relatively stringent *p-*value threshold of 0.01. Our small sample also restricted us from additionally exploring how neural change is influenced by other factors, such as IQ, age sex, home placement status (e.g., foster family, adoptive family, biological or kinship family), severity or type of psychopathology, current medications, and responsiveness to therapy. A second limitation of our research is we did not examine the impact of other prenatal exposures (e.g., nicotine) on our results, even though these may also perturb fMRI results on a Go-NoGo paradigm [106]. Third, we did not monitor Alert^®^ effects outside the lab to see if children who changed most were ones whose parents who applied the therapy at home. Likewise, while all parents at the feedback denied their children received alternative therapies, other forms of stimulation not realized as a therapy may have also influenced our results. Fourth, as we did not include a typically developing or alternate-treatment groups, we do not know whether effects are specific to Alert^®^ or the therapeutic experience more generally. We also do not know what normally happens with repeated presentations of this task and if our observed changes in the untreated FASD group are common to all children tested more than once or unique to children with FASD, particularly as this has never been done in this population. Fifth, since we only obtained a single post-test scan administered shortly the treatment, we do not know whether effects are sustained, thus signifying whether the training is really efficacious. Notably, this is described as a major limitation of most intervention studies on this population [84].

Also, the relatively low accuracy on NoGo trials in a few children may have led to an insufficient number of NoGo trials to model their brain responses. However, since each participant received a total of 90 trials (based on 6 runs with 15 NoGo trials each), we believe this was sufficient for modeling but it may have led to some underpowering, explaining why no few results survived FDR correction. Furthermore, the generally lower accuracy of the WL than TXT group may also have biased the results since WL would have been engaged in more motor activity given they were pressing the response key more frequently. It should be noted, though, that only correct NoGo (i.e., no response) were modeled in the neuroimaging analyses and that accuracy was used as a covariate, thus serving as a proxy for the groups’ different levels of activities.

## 5. Conclusions

This preliminary clinical trial yields positive evidence demonstrating that a treatment targeting a core feature of FASD, namely behavioral dysregulation, yields positive change in critical brain regions underlying the targeted ability. Our results therefore signify an important first step in investigating impact of a clinical therapy on neural outcomes in children with FASD. Importantly, these results also suggest a putative mechanism for behavioral improvement, particularly dampening of fronto-parietal, fronto-cingulate, and fronto-temporal circuits. Overall, the current study lays the groundwork for further more comprehensive and well-controlled trials on this population. Relevantly, too, this research provides hope that effective therapies can have a positive impact on the brain damage caused by prenatal exposure to alcohol.

## Figures and Tables

**Table 1:** Demographic characteristics of the Treatment (TXT) and Waitlist (WL) groups.

Variables	TXT (*N* = 12)	WL (*N* = 13)	*p*-Value
Sex (% male)	50	54	
Age (mean ± SD)	10.3 ± 1.7	10.4 ± 1.3	ns
Birth weight (kg)	2.9 ± 0.68	3.0 ± 0.74	ns
Placement Status:			
% Adopted	75	54	ns
% Fostered	17	8	ns
% With biological relative	8	38	ns
Socioeconomic Status			
% Low	42	69	ns
% Medium	42	8	ns
% High	16	23	ns
Exposure History			
% Alcohol and cigarettes	58	77	ns
% Alcohol and secondary drugs	67	23	0.05
Comorbidities			
% Attention Deficit Hyperactivity Disorder	75	85	ns
% Oppositional Defiant Disorder	25	15	ns
% Learning Disorder diagnosis	50	38	ns
% Anxiety diagnosis	0	15	ns
% Sensory Processing Delay Diagnosis	8	8	ns
% Receiving stimulant medications	67	54	ns
Full Scale IQ (mean ± SD)	86.3 ± 12.7	92.7 ± 15.3	ns

Note: TXT = Alert^®^-treated group; WL = Waitlist control group.

**Table 2:** Mean (±SD) performance data at pre-test and post-test sessions.

Variables	TXT		WL	
	Pre	Post	Pre	Post
Accuracy				
Go trials	0.859 ± 0.14	0.881 ± 0.14	0.893 ± 0.08	0.848 ± 0.08
NoGo trials	0.548 ± 0.20	0.624 ± 0.16	0.430 ± 0.20	0.484 ± 0.19
Go minus NoGo	0.311 ± 0.18	0.257 ± 0.256	0.463 ± 0.16	0.364 ± 0.23
Reaction time (ms) ^a^				
Go trials	0.441 ± 0.09	0.432 ± 0.14	0.400 ± 0.08	0.404 ± 0.11

^a^ Reaction times were not collected on NoGo trials since these involved withholding a response, no responses were made. Note: TXT = Alert^®^-treated group; WL = Waitlist control group.

**Table 3:** Significant findings from whole-brain analyses with the Small-Volume Correction applied.

Contrast		Region BA		MNI Coordinates		Z-Score	FDR *p*-Value	SVC *p*-Value	Cluster Size
***Between-Group Comparisons***									
Waitlist > Treatment	Right medial frontal gyrus	8	8	24	52	2.54	0.350	0.006	12
	Left frontal subgyral	WM	−24	0	32	2.64	0.350	0.004	15
	Right cingulate gyrus	32	16	20	28	2.85	0.350	0.002	30
	Left cingulate gyrus	24	−12	−16	40	2.78	0.350	0.003	12
	Right putamen		24	20	−16	2.99	0.350	0.001	21
	Right superior temporal gyrus	22	56	−56	16	3.19	0.350	0.001	10
***Group X Time Interactions***									
	Right superior frontal gyrus	6	16	12	56	2.75	0.677	0.003	26
	Left middle frontal gyrus	46	−48	40	16	2.58	0.677	0.005	14
	Left inferior parietal lobule	40	−56	−40	52	2.84	0.677	0.002	17
